# Association rare: lichen scléroatrophique extragénital et vitiligo inflammatoire chez un enfant

**DOI:** 10.11604/pamj.2018.30.75.13954

**Published:** 2018-05-29

**Authors:** Ilhame Naciri, Leila Benzekri

**Affiliations:** 1 Service de Dermatologie et Vénérologie, Centre Hospitalier Universitaire IBN SINA, Faculté de Médecine et de Pharmacie, Université Mohammed V, Rabat, Maroc

**Keywords:** Lichen scléro-atrophique, vitiligo, enfant, Scleroatrophic lichen, vitiligo, child

## Abstract

Scleroatrophic lichen (SL) and vitiligo are two depigmenting disorders which may occur separately or, rarely, in combination. Their association may seem logical because both these disorders are characterized by the suspicion of an autoimmune pathogenesis. We here report the case of a 8-year old girl, with no notable medical history, presenting with achromic macules and papular nonpruritic lesions evolving over 6 months. Clinical examination showed two types of lesions (A): ovalaire achromic macules measuring 1-3 cm along their longer axis, located at the level of the front, of the neck, of the shoulders, as well as of the peri-mamelonar and of the genital region. These lesions exhibited slightly raised peripheral inflammatory border as well as a poliosis. The patient also had pearly white atrophic papular plaques at the level of the interscapular and abdominal regions as well as of the anterior face of the knees. Two biopsies were performed. Histological examination of an achromic macula showed vitiligo with inflammatory reaction while histological examination of an infiltrated lesion allowed the diagnosis of scleroatrophic lichen. Significant clinical improvement was obtained by local treatment with dermocorticoids. Therefore, this pathological association between scleroatrophic lichen and vitiligo in our patient, highlights the key role of epidermal lichenoid inflammatory process in the disappearance of melanocytes and, possibly, in the induction of a auto-immune process.

## Image en médecine

Le lichen scléroatrophique (LSA) et le vitiligo sont deux affections dépigmentantes qui peuvent survenir séparément, ou bien rarement coexister. Leur association peut sembler logique en raison de la suspicion d'une pathogénie auto-immune dans les deux entités. Une fillette âgée de 8 ans, sans antécédents pathologiques notables, consultait pour des macules achromiques et des lésions papuleuses non prurigineuses évoluant depuis 6 mois. L'examen clinique objectivait deux types de lésions (A). Des macules achromiques, ovalaires, de 1 à 3 cm de grand axe, situées au niveau du front, du cou, des épaules, ainsi que la région péri-mamelonaire et génitale. Ces lésions présentaient par endroits un liseré inflammatoire périphérique légèrement surélevé, ainsi qu'une poliose. La patiente avait également des plaques papuleuses atrophiques de teinte blanc nacré, localisées au niveau des régions inter scapulaire et abdominale ainsi qu'à la face antérieure des genoux. Deux biopsies ont été pratiquées. L'examen histologique d'une macule achromique était en faveur d'un vitiligo avec réaction inflammatoire et celui d'une lésion infiltrée faisait évoquer le diagnostic de lichen scléroatrophique. Une nette amélioration clinique était obtenue grâce à un traitement local par dermocorticoïdes. Ainsi, cette association pathologique LSA et vitiligo chez notre patiente, met en évidence le rôle déterminant du processus inflammatoire lichénoide épidermique, dans la disparition des mélanocytes, et éventuellement dans l'induction d'un processus auto-immun.

**Figure 1:**
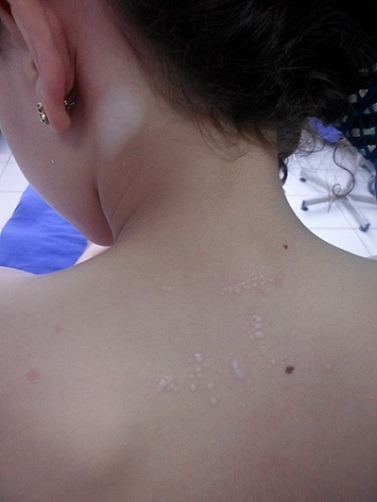
Macule achromique ovalaire de 3 cm de grand axe en regard du cou, associée à des lésions papuleuses atrophiques de couleur blanc nacré, siégeant au niveau de la région inter scapulaire

